# Antioxidant Compounds and Health Benefits of Citrus Fruits

**DOI:** 10.3390/antiox12081526

**Published:** 2023-07-30

**Authors:** Sergio Marques Borghi, Wander Rogério Pavanelli

**Affiliations:** 1 Laboratory of Pain, Inflammation, Department of Pathology, Neuropathy and Cancer, Londrina State University, Londrina 86057-970, Brazil; 2 Center for Research in Health Sciences, University of Northern Paraná, Londrina 86041-140, Brazil; 3 Laboratory of Immunoparasitology of Neglected Diseases and Cancer, State University of Londrina, Londrina 86057-970, Brazil

Recent evidence emanating from epidemiological prospective studies shows that increased intakes of antioxidant-rich fruits, vegetables, and legumes are associated with a lower risk of developing chronic oxidative stress-related diseases like cardiovascular diseases and cancer, as well as with a lower risk of cardiovascular, cancer, and all-cause mortality rates [1,2,3]. Functional food ingredients (also referred to as nutraceuticals) are highly nutritious food-derived products that naturally offer or are modified aiming to promote powerful additional health benefits that go beyond basic nutrition factors. The bioactive compounds present in these dietary items have been extensively studied in recent decades as potential molecules capable of interfering with the pathophysiological mechanisms associate with several diseases. The general benefits provided by the regular consumption of fruits and vegetables are proposed to be conferred by their nutritional compounds, including vitamins, and non-flavonoid and flavonoid polyphenols [4]. Important components of functional foods include citrus fruits produced by the flowering plants from the genus *Citrus* L. (Rutaceae family) [5,6]. Fruits in this group include oranges, mandarin, tangerine, clementine, grapefruit, pomelo, lemons, and lime. Citrus fruits are rich in sugars, vitamins, organic acids (such as hydroxycinnamic, hydroxybenzoic, citric, and succinic acids), coumarins, terpenoids, and flavonoids (including flavanones, flavones, flavonols, and anthocyanins). The biological properties of citrus fruit phytochemicals range from antioxidant and anti-inflammatory to antimutagenic and anticarcinogenic effects, among others [6,7,8].

Oxidative stress denotes a condition provoked by endogenous or exogenous processes in which an imbalance between the generation of free radicals and the cellular ability to neutralize them occurs, thus favoring the overproduction of reactive species. This phenomenon represents a harmful event for cells and tissues, in which the cell membrane, mitochondria and nucleus are highly vulnerable, consequently contributing to the pathogenesis and progression of several diseases [9]. Therefore, targeting oxidative stress in disease has been proposed as a potential approach for diseases prevention and therapy [10]. In this sense, a better comprehension of the mechanisms by which different antioxidants (both natural or synthetic) acts may provide helpful insights and a rationale for successful pharmacological approaches. Antioxidant mechanisms related to citrus fruits compounds are diversified. The inhibition of pro-oxidant enzymes (e.g., xanthine oxidase) and induction of antioxidant enzymes (e.g., catalase, superoxide dismutase, and glutathione peroxidase) [11,12,13], the modulation of redox-sensitive pathways such as nuclear factor κB (NFκB) and nuclear factor E2-related protein 2 (Nrf2) [14,15,16,17,18], reactive oxygen/nitrogen species (ROS/RNS) scavenging [19,20], and the chelation with transition metals [20,21] are some of the effects or actions described for the antioxidant compounds of citrus fruits to combat oxidative stress [6]. Despite these advances, unravelling new potential mechanisms by which citrus fruit-derived compounds may modulate pathological conditions will contribute to bringing new knowledge on their properties and therapeutic applicability. This Special Issue on the “Antioxidant compounds and health benefits of citrus fruits” contains nine contributions, comprising six research articles and three reviews.

In the first original article, Bussmann et al. [17] demonstrated the mechanism by which the synthetic flavonoid hesperidin methyl chalcone (HMC; C_29_H_36_O_15_) protects the kidneys from damage caused by the non-steroidal anti-inflammatory drug (NSAID) diclofenac. HMC is generated via methylation of the flavanone hesperidin (hesperidin-7-rhamnoglucoside) [22]. The data showed that HMC acts by boosting antioxidant parameters and by reducing oxidative damage and pro-inflammatory cytokines both systemically and in renal tissue. In the kidneys, HMC additionally led to an increased production of anti-inflammatory cytokine IL-10 and a reduction in histopathological damage, edema, and the levels of active tubular pathology marker neutrophil gelatinase-associated lipocalin (NGAL), with these effects being attributed to the activation of the Nrf2/antioxidant responsive elements (ARE) pathway [17]. Lai et al. [23] evaluated the effects of five different drying methods (freeze drying, shade drying, hot-air oven drying at 50 °C, hot-air oven drying at 70 °C, and microwave drying) on the bioactive phytochemicals and antioxidant capacity of navel orange peel. Through HPLC analysis, they identified thirteen flavonoids (three flavanone glycosides and ten polymethoxyflavones) in navel orange peel. The authors found that the use of hot-air oven drying at 50 °C or 70 °C for the drying of orange peel delivered the best results, contributing to the maintenance of bioactive compounds in the peel as well as the improvement of its antioxidant capacity, thus advancing the understanding of the useful methods for the viability and antioxidant potential of navel orange peel compounds [23]. Cioni and colleagues [24] conducted comparative chemical analyses among peel and pulp essential oils and methanolic extracts of different *Citrus australasica* varieties (Red, Collette, Pink Ice, and Yellow Sunshine), as well as analyses of the hybrid faustrime (caviar lime). Additionally, the antioxidant activity of peel and pulp extracts using an A31 mouse embryo fibroblast cell line was also investigated. The peels’ essential oils exhibited higher total phenolic contents with greater antioxidant activity. Collette peels showed the highest concentration of flavonoids, including luteolin, isosakuranetin, and poncirin derivatives, and delphinidin and petunidin glycosides. Pink Ice pulps were also shown to be an additional source of flavonoids, and Collette and Red peels presented the highest in vitro antioxidant activity, which was attributed to the presence of anthocyanins, thus identifying finger lime fruits as good sources of phytocompounds within the context of promoting healthy benefits [24]. In another interesting original research article, Ju et al. [25] investigated the antioxidant effects of *Citrus junos* peel fractions (ethanol acetate, hexane, and butanol) using human primary dermal fibroblast and immortalized keratinocyte, and murine melanoma cell lineages. They showed that Yuja peel fractions possessed anti-wrinkle effects by inhibiting metalloproteinases 1, 9, and 13 at mRNA and protein levels, as well as by inducing type I pro-collagen and hyaluronic acid in evaluated UVB-irradiated cells. Moreover, the Yuja peel fractions induced the production of proteins relating to skin hydration, and they decreased melanin content, thus promoting advances in the understanding of the skin benefits provided by Yuja peel fractions, which may contribute to the development of novel pharmaceuticals and cosmetics [25]. In the in vitro study of Nakashima et al. [26], new pharmacological insights into the ways in which high doses of flavonoids quercetin (C_15_H_10_O_7_) and hesperidin (C_28_H_34_O_15_) differentially modulate cell viability, tight junction integrity, and cell shape are provided. Considering that the barrier function of tight junctions may block the absorption of some molecules, the identification of reversible modifiers of its integrity are desirable as drug absorption enhancers [27]. They conclude by suggesting both quercetin and hesperidin are promising compounds for developing a naturally occurring drug absorption enhancer for the paracellular route, with hesperidin being the most attractive for such a technology [26]. Finally, García-Nicolás et al. [28] conducted a spatial metabolomic analysis for the characterization of phenolic compounds in juices and fruit tissue extracts of lemons, limes, and mandarins. Flavonoids were mainly found in the citrus peel (flavedo and albedo) and carboxylic acids in segments, facilitating the extraction of the latter in juices. Limonoids were also distributed in the albedo and segments. The radical scavenging activity was attributed to the flavonoids, and the antioxidant effects were attributed to the combined action of flavonoids and limonoids. These data regarding the fractionation of the extracts advance the comprehension of the antioxidant effects of the family compounds identified [28].

In the first review article, Saini et al. [29] highlighted in their compilation of data the composition and associated health benefits of some components of citrus fruits, notably, carotenoids, flavonoids, limonoids, and terpenes. In their conclusions, the authors propose that bioactive flavonoids of citrus fruits may represent important molecules with antioxidant ant inflammatory properties capable of minimizing the risk of many non-communicable chronic diseases, as well as suggesting that those essential oils rich in limonoids and terpenes possess potential antioxidant and antimicrobial effects. Additionally, the authors pointed to interesting potential future investigations in this field aiming to elucidate some gaps that still exist regarding the composition, content, and health effects of citrus fruit bioactives [29]. In the second review article, Fideles et al. discuss the neuroprotective effects of the flavonol quercetin on nervous system regeneration and functional recovery [30]. Quercetin, one of the most studied and abundant flavonoids in edible vegetables, fruits, and wines, is a pentahydroxyflavone that has hydroxy groups placed at the 3-, 3′-, 4′-, 5-, and 7-positions [31]. The result reported by the authors provided evidence for beneficial effects in preclinical spinal cord injury and peripheral nerve injury models, demonstrating that quercetin can induce effective recovery of neurological functions, contributing to the regeneration of both central and peripheral nervous tissues [30]. Finally, Madureira et al. [32] conducted a comprehensive review regarding the evidence of two antioxidant flavanones, naringenin (C_15_H_12_O_5_) and hesperidin, on the prevention and therapy of breast cancer. Through DNA damage, oxidative stress may trigger genetic alterations that predispose tumorigenicity and tumor progression [33]. The main mechanisms of these flavonoids to counteract breast cancer are properly addressed in the article and are especially associated with anti-proliferative, anti-tumorigenic, and anti-metastatic actions, as well as with the epigenetic modulatory effects upon estrogen receptors [32]. Thus, within effective and safe concentrations, citrus fruits components may represent promising nutraceuticals as anti-cancer substances for breast cancers treatment. 

In conclusion, the original research and review articles address several interesting experimental conditions aiming to explore the antioxidant potential of citrus fruit compounds in the context of health benefits. The progress regarding the best methods for its utilization, the elucidation of the mechanistic actions of each bioactive, and the identification of the most effective doses, with guaranteed safety of each antioxidant compound of citrus fruits, will help to develop a rationale to obtain advanced technology which will be useful to optimize its beneficial effects. 
